# PVA-KH792-Enhanced Composite Cementitious Material from Lead–Zinc Slag and Electroplating Sludge: Mechanical Performance and Heavy-Metal Immobilization

**DOI:** 10.3390/ma19071420

**Published:** 2026-04-02

**Authors:** Pengpeng Zhang, Dongwei Li

**Affiliations:** 1 State Key Laboratory of Coal Mine Disaster Dynamics and Control, Chongqing University, Chongqing 400044, China; zpp2017cqu@163.com; 2 College of Resources and Safety Engineering, Chongqing University, Chongqing 400044, China

**Keywords:** lead and zinc smelting slag, polyvinyl alcohol, silane coupling agent KH792, electroplating sludge, immobilization of heavy metals

## Abstract

**Highlights:**

- LZSS and electroplating sludge were used to prepare an alkali-activated cementitious material.
- PVA-KH792 co-modification improved compressive strength and reduced heavy-metal leaching.
- Heavy metals are immobilized via redox, chemical bonding/chelation, and physical encapsulation.
- This work offers a potential approach to transform electroplating sludges and LZSS into non-structural building materials.

**Abstract:**

To address the limited simultaneous optimization of mechanical performance and heavy-metal stabilization in waste-based alkali-activated systems, this study investigates the development and characterization of a novel composite cementitious material for potential construction applications, utilizing lead and zinc smelting slag (LZSS) and electroplating sludge (ES) as precursors. The novelty of this study lies in the co-modification of an LZSS-based alkali-activated matrix with PVA and KH792 to improve both compressive behavior and heavy-metal stabilization in ES-containing specimens. Based on single-factor optimization, the optimal matrix was obtained at 3.5% alkali content, a water-glass modulus of 1.4, and a liquid-to-solid ratio of 0.22, followed by 28 days of curing before testing. On this basis, ES and PVA-KH792 were introduced to investigate their effects on mechanical behavior, heavy-metal leaching, and immobilization mechanisms. The results showed that adding ES reduced the compressive strength of the alkali-activated matrix, whereas PVA-KH792 modification partially restored matrix integrity and improved performance. At 5% ES content, the compressive strength of the modified specimen increased by 7.66% compared with that of the unmodified ES-containing sample. More importantly, under the sulfuric acid–nitric acid leaching method, the Cr leaching concentration decreased from 20.1 mg/L to 13.7 mg/L, meeting the relevant regulatory limit (GB5085.3-2007 and EPA limit). Microstructural and spectroscopic analyses indicated that the beneficial effect of PVA-KH792 was associated with matrix densification and enhanced heavy-metal immobilization. The immobilization mechanisms were mainly attributed to Cr(VI) reduction by Fe(II), complexation/coordination with functional groups introduced by PVA-KH792, and physical encapsulation within the alkali-activated matrix. The findings provide a promising approach to waste valorization and the development of sustainable building materials, contributing to resource efficiency and reducing the environmental impact of the construction sector.

## 1. Introduction

Lead and zinc are critical non-ferrous metals that play a crucial role in industrial production and people’s daily lives. China is a significant producer of non-ferrous metals, accounting for 47% and 35% of the global lead and zinc production, respectively [1]. However, the production process generates substantial waste: approximately 7100 tons of residue per 10,000 tons of lead and 9600 tons per 10,000 tons of zinc [2]. This waste, known as lead and zinc smelting slag (LZSS), accumulates over time and poses significant environmental risks due to heavy-metal leaching, particularly when improperly managed [3].

Solidification techniques can effectively reduce the risk of heavy-metal release into the environment from hazardous waste [4]. Over the years, various solidification methods have been developed, among which the immobilization of heavy metal-containing inorganic materials in mortar and concrete produced with Ordinary Portland Cement (OPC) is a practical approach [5]. However, the OPC production process may generate significant carbon dioxide emissions, detrimental to environmental protection [6]. Converting hazardous waste into glass or glass-like materials for immobilizing heavy metals is an effective technique, but it requires substantial energy consumption during treatment [7]. In comparison, alkali-activated geopolymer technology offers a lower carbon footprint and energy consumption. It has a simple process that only requires materials containing silica–alumina sources and an alkaline activator to produce [8]. The resulting cementitious materials, delivered through geopolymerization reactions, consist of [SiO_4_] and [AlO_4_] units interconnected by covalent bonds in a three-dimensional structure, with the charge balanced by alkali cations [9]. The formation of geopolymer materials generally undergoes several steps [10,11,12]. Firstly, the amorphous components of the raw materials are eroded in the alkaline solution, leading to the rupture of Si-O and Al-O bonds, the gradual dissolution of the raw materials, and the release of a large number of silicon–aluminum tetrahedra. Subsequently, the silicon–aluminum tetrahedra combine to form alternating secondary tetrahedral rings. Charge neutralization occurs by generating voids within the structure, creating a low-polymerized gel. The low-polymerized gel undergoes minor adjustments and interconnections over a small range, gradually forming a complete three-dimensional network. Finally, the system undergoes further condensation reactions and the removal of free water, forming a dense geopolymer. Therefore, employing geopolymer technology for treating lead–zinc smelting slag is an environmentally friendly and energy-efficient approach. This process aligns with cleaner production principles by reducing waste, conserving resources, and minimizing environmental impact.

Some studies have used LZSS as the sole silica–alumina source to prepare geopolymer materials or as a precursor for immobilizing heavy metal-rich hazardous waste [13,14]. Most research on LZSS involves the addition of supplementary cementitious materials containing active silica–alumina sources [3,15,16,17], possibly because LZSS has a relatively low silica–alumina content, which is commonly believed to confer lower geopolymerization activity. However, some studies suggest that iron participates in the geopolymerization process and enters the silicoaluminate network in LZSS [18,19], which may explain why iron-rich LZSS can serve as the sole silica–alumina source for geopolymer material preparation. Additionally, it has been found that LZSS contains both Fe(II) and Fe(III) states [20]. The presence of Fe(II) in LZSS creates a reducing environment during alkali activation, which may aid in detoxifying heavy metals like Cr(VI). Previous studies have demonstrated the feasibility of LZSS-based alkali-activated binders, but many require supplementary reactive precursors and rarely examine polymer-assisted heavy-metal stabilization. Accordingly, the combined use of PVA and KH792 in an Fe-rich LZSS/ES system remains insufficiently explored.

Polymers are commonly used as additives in construction materials and possess various functionalities, including enhancing the compressive strength, durability, and corrosion resistance of cementitious systems [21,22,23]. Polyvinyl alcohol (PVA) is a non-toxic, biodegradable polymer with high chemical resistance and adhesive strength, often used as a cementitious additive, emulsifier, and dispersant [24]. PVA contains numerous hydroxyl groups (-OH) that facilitate the formation of polymer networks through hydrogen bonding [25]. In treating heavy-metal wastewater, PVA is often used to produce hydrogels that can adsorb heavy metals through the functional groups on their surfaces [26,27]. PVA has been widely used as a reinforcement in geopolymers. However, existing studies mostly employ PVA in fiber form [28,29,30,31,32], with fewer instances of its utilization in powdered or water-soluble states. The silane coupling agent contains organic functional and alkoxysilane groups in a single molecule. The alkoxysilane groups hydrolyze to form Si-OH, which can crosslink with the -OH groups on PVA [33] and form covalent bonds with the inorganic surface. This phenomenon can effectively improve the adhesion performance at the inorganic/polymer interface [34]. KH792 ([3-(2-Aminoethyl)aminopropyl] trimethoxysilane) is a bifunctional amino silane, and its modification can enhance the adsorption of heavy-metal ions by materials [35]. PVA and KH792 were combined because PVA can form a polymer network rich in hydroxyl groups, whereas KH792 can enhance organic–inorganic interfacial bonding and provide amino functionalities; together, these features may favor both matrix densification and heavy-metal immobilization. In the present LZSS-based system, the modifier combination was selected because ES addition weakens the matrix. At the same time, the hydroxyl/amino/silanol functionalities of PVA and KH792 may help reinforce the interface and promote heavy-metal retention. By integrating these additives, this research aims to develop a geopolymer composite that not only exhibits superior mechanical strength but also effectively immobilizes heavy metals from electroplating sludge (ES), a hazardous waste containing Pb, Cr, and Cu.

In response to the pressing challenges of industrial waste management and the increasing demand for sustainable construction materials, this study investigates a novel composite cementitious material derived from lead–zinc smelting slag (LZSS), strategically modified with polyvinyl alcohol (PVA) and the silane coupling agent KH792. The potential of this composite cementitious material to concurrently enhance mechanical performance while effectively immobilizing hazardous heavy metals (Pb, Cr, Cu) originating from co-incorporated electroplating sludge (ES) is systematically evaluated. Accordingly, the primary objectives encompass (1) quantitatively assessing the influence of PVA-KH792 modification on the composite’s compressive strength and heavy-metal leachability, and (2) elucidating the fundamental mechanisms governing heavy-metal immobilization within this developed material matrix. We hypothesized that the co-addition of PVA and KH792 would provide a synergistic effect in the LZSS-based alkali-activated system by enhancing matrix densification and interfacial bonding, while also improving heavy-metal immobilization through structural encapsulation, functional-group interaction, and Fe(II)-assisted Cr(VI) reduction.

## 2. Materials and Methods

### 2.1. Materials

The LZSS used in this experiment was obtained from a lead–zinc smelting plant in Yunnan, China. The ES was sourced from an environmental organization in Chongqing. The oxide compositions of LZSS and ES were determined using X-ray fluorescence analysis, as shown in Table 1 and Table 2, respectively. LZSS contains a small amount of Zn, while ES contains heavy metals such as Pb, Cr, and Cu. Pre-activation phase characterization by XRD indicated that LZSS contains an amorphous glassy phase with hedenbergite/wustite-related features, whereas ES mainly contains lead chromate and CaSO_4_.

The original samples were tested using the Toxicity Characteristic Leaching Procedure (TCLP) and the HJ/T299-2007 method (sulfuric–nitric acid method) [36], and the leaching concentrations of heavy metals are presented in Table 3. The leaching concentrations of all heavy-metal elements in LZSS, except for zinc, are significantly lower than the limits specified by the EPA (U.S. Environmental Protection Agency) relevant standard 1311 [37] and GB5085.3-2007 [38]. The leaching concentration of zinc in the TCLP leachate is 57.2 mg/L. Therefore, in LZSS-solidified materials, only zinc leaching needs to be considered. ES contains substantial amounts of heavy metals and is a highly hazardous material, primarily containing copper (Cu), zinc (Zn), and chromium (Cr). Cu and Cr leaching concentrations exceed the specified limits by a significant margin. The toxicity of hexavalent chromium (Cr(VI)) is 100 times higher than that of trivalent chromium (Cr(III)), and it has carcinogenic effects on the human body. The Cr(VI) leaching concentration in the TCLP method is 69 times higher than the specified limit. In comparison, the leaching concentration of Cr(VI) in the sulfuric–nitric acid method is 50 times higher than the specified limit.

The water glass (sodium silicate solution) was purchased from YouSuo Company (Linyi, China), with a modulus of 3.3 and a solute mass fraction of 34.8%. Analytical grade NaOH was sourced from Chuandong Company (Chongqing, China). PVA was purchased from Inner Mongolia Shuangxin Company (Ordos, China), and its physicochemical properties are shown in Table 4. The silane coupling agent KH792 was obtained from YouSuo Company (Linyi, China).

### 2.2. Experimental Method

#### 2.2.1. Preparation of LZSS Samples

The dried coarse LZSS was milled in a ball mill at 200 rpm for 20 h. Subsequently, the milled material was sieved through a 200-mesh sieve to obtain the alkali-activated raw material. The particle size distribution of the sieved sample was mainly concentrated in the 10–100 μm range, with an average particle size of 68.79 μm and a median of 55.83 μm.

The water glass (sodium silicate solution, modulus = 3.3) used as the alkaline silicate source, NaOH was added to adjust the activator composition to the target modulus and alkali content. Deionized water was added to achieve the target liquid-to-solid ratio specified in Table 5 (0.20–0.28, depending on the experimental group). After mixing NaOH, water glass, and deionized water, the alkali activator was allowed to equilibrate at room temperature for 20 h before use.

The ball-milled LZSS was dried in an oven at 105 °C, then poured into the alkali activator and stirred. After achieving a uniform mixture, a thick and dense slurry was obtained. The slurry was then poured into a steel mold and vibrated on a vibration table to remove air bubbles. The initial curing condition for the first 24 h was 30 °C and a relative humidity of at least 90%. After 24 h, the specimens were demolded and subsequently cured in air at room temperature (25~30 °C) away from direct sunlight. This study conducted a single-factor experimental investigation with alkali content, modulus of water glass, and liquid-to-solid ratio as variables. The specific parameters are presented in Table 5.

#### 2.2.2. ES Dosage

Based on the single-factor experiment, the sample with the highest compressive strength was selected as the base, and ES was added. The ball-milled ES was dried in an oven at 105 °C. Different proportions (5%, 7.5%, 10%, 20%, 30%) of ES were substituted for LZSS at equivalent levels during curing. The preparation procedure was identical to that described in Section 2.2.1. The particle size distribution of the ES was mainly in the 10–100 μm range, with an average particle size of 65.31 μm and a median of 53.24 μm.

#### 2.2.3. PVA/KH792 Reinforcement

Different mass fractions (0.5%, 1%, 1.5%, 2%) of PVA and KH792 were added to the cementitious material, and the optimal ratio was determined. The PVA and KH792 at the optimal ratio were then incorporated into the composite cementitious material with a specific ES content, following the same preparation method described in Section 2.2.1. Before use, PVA was dissolved in deionized water, while KH792 underwent pre-hydrolysis. The mixture of PVA and KH792 formed PVA-KH792.

#### 2.2.4. Leaching Test

Leaching toxicity experiments were carried out using the US EPA (TCLP) and the HJ/T299-2007 (sulfuric–nitric acid method). The 28-day cured specimens were crushed to a particle size below 9.5 mm for the experiments; each specimen had three parallel samples. In the TCLP test, a leaching solution of acetic acid with a pH of 2.88 ± 0.05 was used, and the leaching solution and solid particles were mixed at a liquid-to-solid ratio of 20:1 (L/kg) for 18 ± 2 h. Upon completion, vacuum filtration was performed, and the heavy-metal content of the collected leaching solution was determined by atomic absorption spectrometry. The sulfuric–nitric acid method is similar to TCLP, with the only difference being the use of sulfuric and nitric acids as the leaching solution at a 2:1 mass ratio. The pH was adjusted to 3.20 ± 0.05 with deionized water, and the liquid-to-solid ratio was set at 10:1 (L/kg).

### 2.3. Characterization Methods

**Figure 1:**
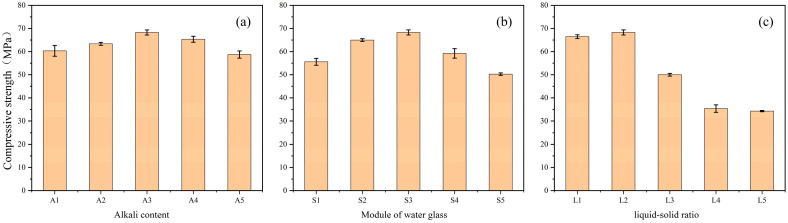
Compressive strength results of single-factor experiment: (**a**) alkali content; (**b**) water glass modulus; (**c**) liquid–solid ratio.

**Figure 2:**
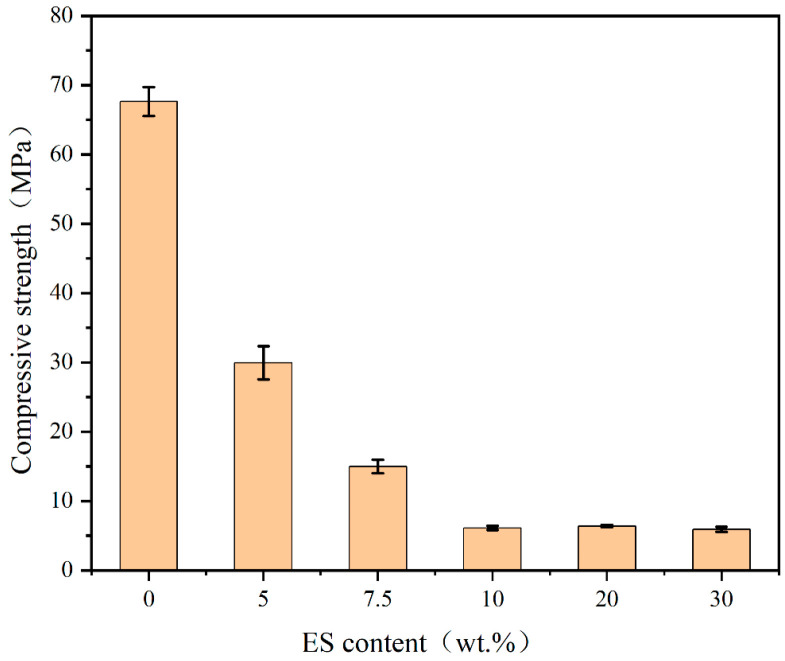
Compressive strength results of different ES content.

**Figure 3:**
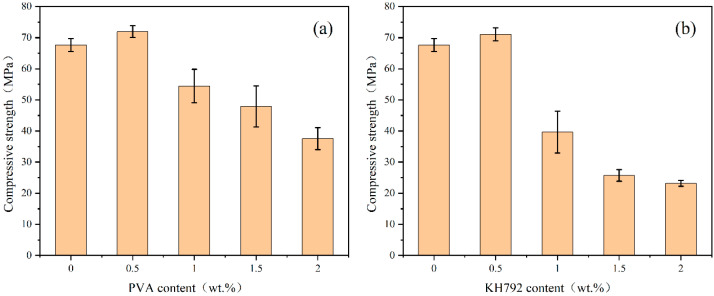
(**a**) Compressive strength of PVA-LZAC; (**b**) compressive strength of KH792-LZAC.

**Figure 4:**
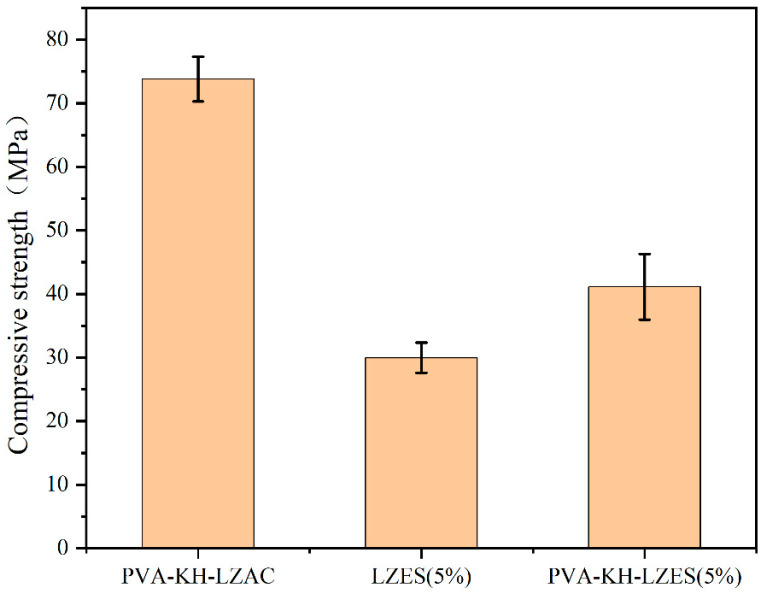
Compressive strength of PVA-KH792 and ES incorporation.

The compressive strength of the samples was tested in accordance with GB/T17671-1999 [39] and ASTM C1948/C1948M-24 [40]. The compressive strength tests were conducted using a multifunctional testing machine (AGN-250, Shimadzu, Kyoto, Japan). Each sample was prepared in triplicate and tested after 28 days of curing, and the average value was used as the final result. The error bars with standard deviation are presented in Figure 1, Figure 2, Figure 3 and Figure 4. The specimen size was 2 cm × 2 cm × 2 cm. The testing machine had a maximum capacity of 125 kN, and a uniaxial compressive load was applied uniformly to the specimen at a rate of 1 mm/min to obtain the compressive strength.

X-ray fluorescence (XRF) was employed to determine the elemental composition of the raw materials. X-ray diffraction (XRD) analysis was performed to examine the crystal structure under the following conditions: CuKα radiation, 40 kV, 30 mA, 10~90°, 2°/min. Fourier-transform infrared spectroscopy (FTIR) was used to investigate the samples’ molecular structure and chemical bonds under the following conditions: ART, 400~4000 cm^−1^, 4 cm^−1^ resolution. Scanning electron microscopy with energy dispersive spectroscopy (SEM-EDS) was employed for microstructure observation and analysis of micro-area composition. Magnification varied across samples; specific magnifications are indicated in the legends of the SEM images. X-ray photoelectron spectroscopy (XPS) was used to analyze chemical bonds and atomic valence states. XPS spectra were processed after background subtraction and peak deconvolution using a Gaussian–Lorentzian fitting method.

## 3. Results

### 3.1. Compressive Strength

#### 3.1.1. Single Factor Experiment of LZSS

The alkali content refers to the ratio of the relative molecular mass of the base to the number of hydroxyl groups contained in the molecule [14]. This experiment set the alkali content at five levels (2.5%, 3%, 3.5%, 4%, 4.5%). As shown in Figure 1a, the compressive strength of the samples first increases and then decreases with increasing alkali content. During the geopolymerization reaction, the silicoaluminate oxide dissolves in the alkaline solution [41]. As the alkali content increases, the dissolution rate increases within a specific range, allowing sufficient dissolution of silicoaluminate raw materials and thereby increasing compressive strength. However, excessively high alkali content limits ion migration and reduces the compressive strength of the geopolymer paste [42].

Water glass, also known as alkali metal silicate, is a compound with the chemical formula R_2_O · nSiO_2_. In this experiment, sodium water glass was utilized. The modulus of water glass refers to the molar ratio of SiO_2_ to Na_2_O. It is also known as the silica or silicic acid modulus (the modulus of water glass used in this experiment is 3.3). Soluble silicate plays a crucial role in the geopolymerization process. It provides soluble silicate to the aqueous phase of the geopolymer system, which is necessary for oligomer formation. As depicted in Figure 1b, an increase in the water glass modulus increases the soluble silicate content in solution, thereby enhancing geopolymerization efficiency [43]. However, when the water glass modulus is excessively high, the NaOH concentration in the solution decreases, resulting in insufficient dissolution of the silicon–aluminum oxide. Then hinders the complete participation of raw materials in the reaction [44].

According to Figure 1c, the compressive strength of the samples initially increases and then decreases with increasing liquid-to-solid ratio. When the liquid-to-solid ratio is 0.18, the sample’s hydration degree is too low, making it difficult to form a solid structure. The lower compressive strength at a lower liquid-to-solid ratio may be due to insufficient water content, which prevents complete dissolution of the raw materials and hinders the progress of the geopolymerization reaction. On the other hand, when the liquid-to-solid ratio is too high, although it improves the flowability of the sample, it also prolongs the setting time, and an excess amount of water could potentially impede the polymerization process and the establishment of a stable network configuration [45], resulting in a decrease in compressive strength. Considering all factors, the highest compressive strength of 68.28 MPa was achieved with an alkali content of 3.5%, a water glass modulus of 1.4, and a liquid-to-solid ratio of 0.22.

#### 3.1.2. Experiment of ES Dosage

From Figure 2, it can be observed that as ES is added, the compressive strength of the samples decreases continuously. This phenomenon can be attributed to two factors. Firstly, adding sludge may consume some NaOH, leading to incomplete dissolution of the raw materials. Secondly, the significant decrease in compressive strength may be due to heavy metals in the ES, which damage the three-dimensional network structure of the samples [46].

#### 3.1.3. Organic Matter Enhanced LZAC/LZES

From Figure 3a, it can be observed that the compressive strength of the samples initially increases with PVA addition, then decreases continuously. At a 0.5% dosage, the sample’s compressive strength increased from 68.28 MPa to 71.93 MPa, indicating a 5.35% improvement. However, the increase in compressive strength is not significant, which may be attributed to the formation of a film when PVA powder dissolves in water, which hinders the geopolymerization reaction to some extent [47]. This effect becomes more pronounced at higher PVA dosages.

In the experimental group with the addition of KH792 (Figure 3b), the change in compressive strength follows a similar trend: initially increasing and then decreasing with increasing KH792 dosage, reaching a maximum at 0.5%. However, the increase in compressive strength from KH792 addition was not significant. This phenomenon may be attributed to two factors. Firstly, the hydrolysis of the silane coupling agent, KH792, leads to Si-OH formation, which then combines with the Si-O in [SiO_4_], thereby slowing down the geopolymerization reaction to a certain extent. Secondly, adding the silane coupling agent may increase the water content of the mixture, which can also affect the compressive strength. A comparison with our previous studies on the preparation of alkali-activated cementitious materials from lead–zinc smelting slag indicates that the addition of PVA and KH792 both enhances the compressive strength of LZSS-based cementitious materials [3,13,14].

The optimal dosage of 0.5% for both PVA and KH792 was determined. A mixture of 0.5% PVA and 0.5% KH792, referred to as PVA-KH792, was prepared and added to the samples, resulting in an increased compressive strength of 73.79 MPa (Figure 4). The obtained sample exhibited a 7.66% improvement compared to LZAC alone and also outperformed samples with only 1% PVA or 1% KH792. This phenomenon indicates a synergistic effect between PVA and KH792, enabling a better combination with the cementitious material when they are co-added. According to the results in Section 3.1.2, a 5% mass fraction of ES was selected for mechanical property studies. It was found that the compressive strength of LZES decreased to 29.95 MPa after adding 5% ES (Figure 4), but increased to 41.12 MPa after the composite addition of PVA-KH792 (Figure 4). A comparison with our prior research on the stabilization of heavy metals using cementitious materials derived from lead–zinc smelting slag shows that, at ES dosages of 3%, 5%, and 7.5%, the compressive strength of the prepared samples remains above 10 MPa, demonstrating superior performance compared to earlier results [13,16,20]. This can be attributed to the formation of a three-dimensional network structure by PVA itself, which, under the action of the silane coupling agent, partially interconnects with the cementitious material. Additionally, the -OH groups in PVA and the NH2 groups in KH792 can adsorb and chelate some heavy-metal ions, thereby reducing their detrimental effects on the three-dimensional network structure formed during geopolymerization.

### 3.2. Leaching Experiment

For samples cured for 28 days, leaching toxicity tests were conducted following the methods described in Section 2.2.2. The test results are presented below.

#### 3.2.1. The Leaching of Zn

The leaching concentration of zinc, a heavy metal, in LZSS without ES addition is shown in Table 6.

Experimental results showed that the leaching concentration of Zn in LZSS decreased from 57.2 mg/L (TCLP) and 0.82 mg/L (sulfuric–nitric acid method) to 46.31 mg/L (TCLP) and 0.43 mg/L (sulfuric–nitric acid method) after alkaline activation curing. With the addition of PVA, the leaching concentration of Zn initially increased, then decreased, suggesting a possible relationship between Zn fixation and physical encapsulation. In the groups with the addition of the silane coupling agent, the leaching concentration of Zn decreased slightly with increasing KH792 dosage, suggesting the formation of Zn-KH792 complexes for fixation. Upon the composite addition of 0.5% PVA and 0.5% KH792, the leaching concentration of Zn decreased to 31.03 mg/L (TCLP), lower than the individual addition of 1% PVA, 43.92 mg/L (TCLP), or 1% KH792, 45.37 mg/L (TCLP). This phenomenon indicates a synergistic effect between PVA and KH792.

#### 3.2.2. The Leaching of Pb, Cr, Cu

**Figure 5:**
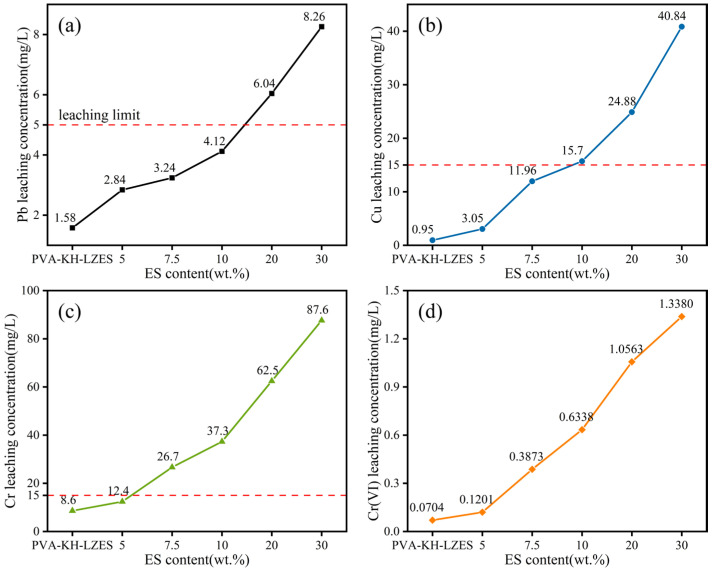
TCLP heavy-metal leaching concentration: (**a**) Pb leaching concentration; (**b**) Cu leaching concentration; (**c**) Cr(III) leaching concentration; (**d**) Cr(VI) leaching concentration; the red dotted line is the limit value of leaching concentration.

Figure 5 presents the TCLP leaching concentrations of heavy metals. It can be observed that the leaching concentrations of different heavy metals increase with increasing ES dosage. As depicted in Figure 5a, the Pb leaching concentration remains below the limit when the ES dosage is below 10%. Similarly, Figure 5b shows that the leaching concentration of Cu is below the limit when the ES dosage is below 7.5%. Furthermore, Figure 5c demonstrates that the leaching concentration of total Cr is below the limit at an ES dosage of 5%. Additionally, Figure 5d shows that the Cr(VI) leaching concentration in all ES dosage groups is below the limit. These results suggest that the free Cr(VI) is effectively immobilized during the alkaline activation process.

**Figure 6:**
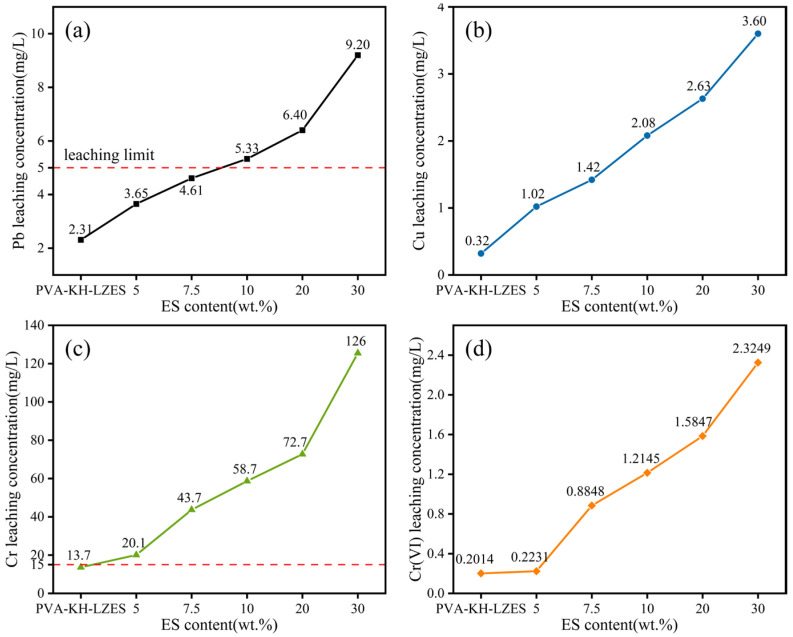
Leaching concentration of heavy metals by sulfuric acid-nitric acid method: (**a**) Pb leaching concentration; (**b**) Cu leaching concentration; (**c**) Cr(III) leaching concentration; (**d**) Cr(VI) leaching concentration; the red dotted line is the limit value of leaching concentration.

Figure 6 represents the leaching concentrations of heavy metals in the sulfuric–nitric acid method samples. Similarly to the trend observed in TCLP leaching concentrations, heavy-metal leaching concentrations also increase with the addition of ES. In Figure 6a, the Pb leaching concentration remains below the limit when the ES dosage is below 7.5%. Figure 6b shows that the leaching concentration of Cu is below the limit value for all ES dosage groups. In Figure 6c, the leaching concentration of total Cr remains above the limit value at an ES dosage of 5%. Figure 6d demonstrates that Cr(VI) leaching concentration is below the limit value for all ES dosage groups. Notably, the leaching concentration of Cu in the sulfuric–nitric acid method is significantly lower than that in the TCLP method (Figure 6b). This phenomenon may be attributed to the higher initial pH (3.20 ± 0.05) of the leaching solution in the sulfuric–nitric acid method compared to the TCLP leaching solution (2.88 ± 0.05), which causes partial precipitation of Cu(II) during the leaching process. Apart from Cu, the leaching concentrations of other heavy-metal ions are higher than those obtained by the TCLP method, possibly due to the lower liquid-to-solid ratio in the sulfuric–nitric acid method.

Based on the leaching concentrations of heavy metals obtained using the TCLP and sulfuric–nitric acid methods, a 5% ES dosage for the cementitious material was determined. However, at this dosage, the Cr leaching concentration in the sulfuric–nitric acid method still exceeded the limit of 15 mg/L, rendering the samples non-compliant with the standards. Figure 5 and Figure 6 also display the results of the leaching concentrations of heavy metals with the addition of PVA-KH792 at a 5% ES dosage. It was observed that the addition of PVA-KH792 decreased the leaching concentrations of heavy metals, specifically the Cr concentration in LZES using the sulfuric–nitric acid method, from 20.1 mg/L to 13.7 mg/L, ensuring that the samples complied with the specified leaching concentration limits. Although laboratory leaching compliance was achieved at 5% ES after PVA-KH792 modification, field deployment would still require long-term durability and environmental validation.

Overall, the results reveal a close relationship between compressive strength development and heavy-metal leaching behavior in the LZSS-based alkali-activated system. The single-factor optimization results show that appropriate alkali content, water-glass modulus, and liquid-to-solid ratio promote geopolymerization and yield a mechanically stronger matrix, which also provides a more favorable structural basis for heavy-metal immobilization. As ES content increased, compressive strength decreased gradually. In contrast, the leaching concentrations of some heavy metals increased, indicating that incorporating hazardous sludge disrupted matrix continuity and weakened the solidified body’s retention capacity. In contrast, after PVA-KH792 co-modification, the compressive strength was recovered or further improved, and the leaching concentrations of Zn and Cr were correspondingly reduced. In particular, at 5% ES content, the Cr leaching concentration decreased to below the hazardous-waste identification limit, suggesting that the co-modified system not only improved mechanical integrity but also enhanced the solidification/stabilization capacity toward heavy metals. These results indicate that a denser, more integrated matrix is generally associated with a lower leaching risk, and that the synergistic action of PVA and KH792 is an important factor in achieving simultaneous improvements in strength and heavy-metal immobilization.

## 4. Discussion

### 4.1. Characterization Analysis

#### 4.1.1. XRD Analysis

**Figure 7:**
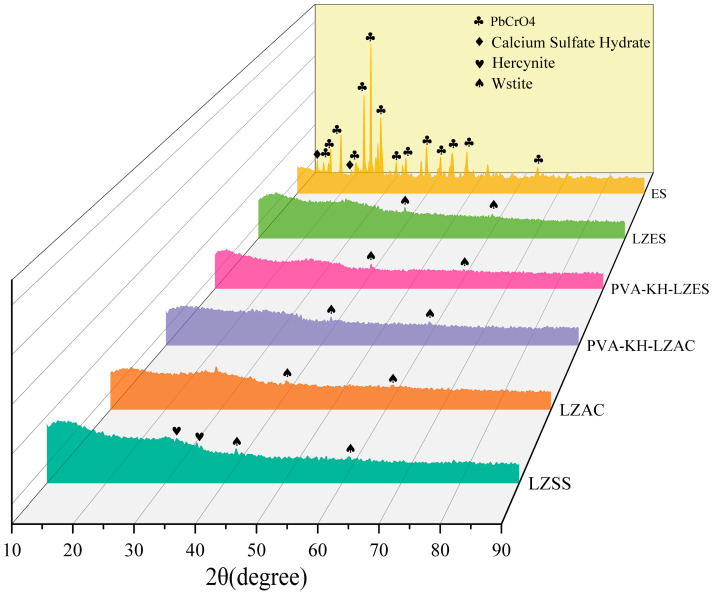
XRD spectra of LZSS, ES, LZAC, LZES, PVA-KH-LZAC, and PVA-KH-LZES.

The XRD spectrum of LZSS is shown in Figure 7. From the graph, it can be observed that LZSS exhibits broad diffraction peaks between 10° and 20° as well as 25° and 40°, indicating the presence of an amorphous glassy phase. Previous studies have suggested that amorphous phases are conducive to geopolymerization reactions [48,49], indicating that LZSS possesses some geopolymerization activity. In addition to the amorphous diffraction peaks, hedenbergite and wustite minerals are also observed, with LZSS appearing black–green, indicating a relatively high Fe(II) content. The disappearance of the hedenbergite crystalline peak after alkaline activation suggests that NaOH may alter the structure of hedenbergite [50]. In LZAC, broadening the diffraction peaks between 25° and 40° is associated with the formation of C-S-H and N-A-S-H phases resulting from alkaline geopolymerization reactions [13]. No new crystalline phases were observed in samples containing PVA-KH792, suggesting that organic substances do not participate in the formation of crystalline phases in the cementitious material. ES mainly consists of lead chromate (PbCrO_4_·xPbO) and contains some CaSO_4_. However, no crystalline lead chromate peaks were detected in samples with ES, suggesting a change in the chemical form of the heavy metals, possibly due to the dissolution of lead chromate in the alkaline solution. A slightly weaker crystalline peak of magnetite was observed in LZES and PVA-KH-LZES compared to LZAC. Considering the changes in the leaching concentration of Cr(VI), some Fe(II) ions from LZSS dissolved in the alkaline solution may reduce Cr(VI) to Cr(III) during the alkaline activation process.

#### 4.1.2. FTIR Analysis

**Figure 8:**
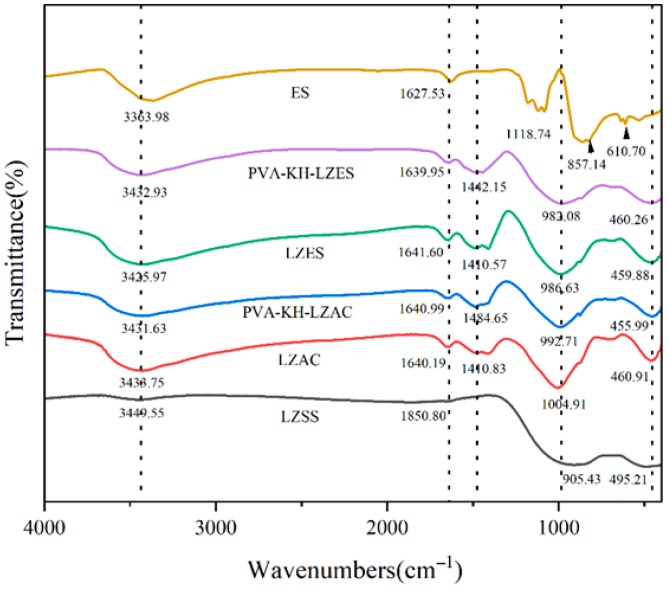
FTIR spectra of LZSS, LZAC, PVA-KH-LZSS, LZES, PVA-KH-LZES, and ES.

As shown in Figure 8, regardless of the addition of PVA or KH792, the chemical bonds in all cementitious materials remain unchanged, as indicated by their FTIR spectra, which are generally similar, with only some changes in wave numbers. The characteristic peaks around 3363–3449 cm^−1^ and 1600 cm^−1^ correspond to the stretching vibration of -OH and the bending vibration of H-O-H, respectively, which may be due to the presence of OH groups in the hydrated aluminosilicate formed during alkaline activation and adsorption of a small amount of moisture on the sample surface [51,52]. The peaks between 1410 and 1484 cm^−1^ correspond to the stretching vibration of O-C-O in CO_3_^2−^, indicating the carbonation of alkali metal hydroxides in the samples to form carbonates [3]. It is worth noting that LZSS has higher Fe content than kaolin- or fly ash-based cementitious materials. Some studies have suggested that Fe may be incorporated into the three-dimensional network structure of cementitious materials [53]. Hence, the absorption peak observed between 900 and 1000 cm^−1^ is attributed to the asymmetric stretching vibration of Si-O-T (T = Si, Fe, Al). It has been found that the fixation effect of heavy metals is mainly based on Si-O-T [54]. Compared to LZAC, the absorption peaks in samples with ES shift to lower wavenumbers, possibly due to the transformation of Si-O-T into non-bridging oxygen-bonded Si-O-M (M = Na, Ca) after alkali reaction with the aluminosilicate [14], thereby generating exchangeable sites. Heavy-metal ions replace Ca^2+^ and Na^+^ and participate in the geopolymerization reaction, supporting a similar conclusion by Hu [55]. The absorption peak near 460 cm^−1^ corresponds to the bending vibration of Si-O bonds [56]. In the ES spectrum, the peaks around 1118 cm^−1^ and 610 cm^−1^ correspond to the absorption of SO_4_^2−^. Due to the relatively low SO_4_^2−^ content, the absorption peak at 610 cm^−1^ is less pronounced and may be affected by other functional groups. The peak near 857 cm^−1^ corresponds to the symmetric stretching vibration mode of Cr-O [57].

#### 4.1.3. SEM-EDS Analysis

**Figure 9:**
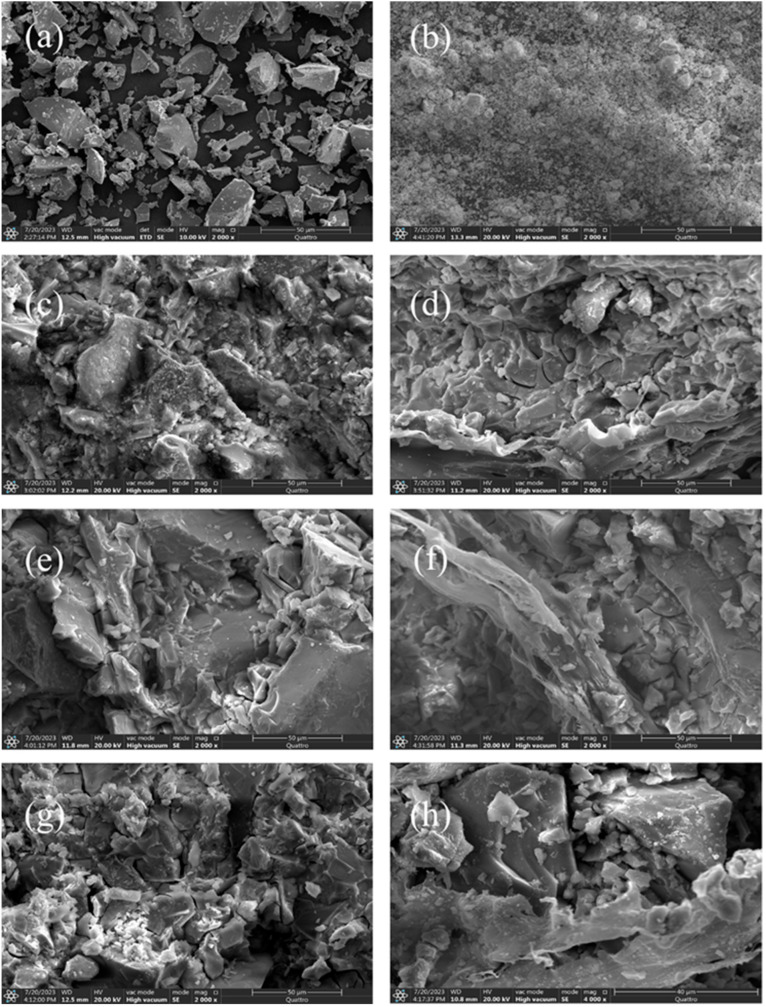
SEM images of (**a**) LZSS; (**b**) ES; (**c**) LZAC; (**d**) PVA; (**e**) KH792; (**f**) PVA-KH-LZAC; (**g**) LZES; and (**h**) PVA-KH-LZES.

Figure 9 presents the SEM images of the raw materials and samples. From the SEM images of LZSS (Figure 9a) and ES (Figure 9b), it can be observed that LZSS consists of irregular, block-like particles of varying sizes, with distinct edges and corners. At the same time, ES appears as powdered particles of varying sizes. In the SEM image of LZAC (Figure 9c), a relatively dense structure is observed, with partially unreacted particles encapsulated by the C-S-H or N-A(F)-S-H gel formed during alkaline activation. Figure 9d shows that PVA powder dissolves in water and, after drying, forms a film that tightly binds to the cementitious material matrix through hydrogen bonding. In Figure 9e, fewer microcracks are observed in the matrix, which may be attributed to the condensation reaction between the hydrolyzed Si-OH in KH792 and the O-Si-O in LZSS, leading to the combination of KH792 with the cementitious material and thus filling some cracks. Figure 9f shows the co-addition of PVA and KH792 to the cementitious material, resulting in a denser structure. This phenomenon could be due to the crosslinking between KH792 and PVA, leading to their chemical bonding with the cementitious material. From Figure 9g, it is evident that the addition of ES to LZSS significantly damages the structure of the cementitious material. The inclusion of ES consumes some NaOH solution, inhibiting the geopolymerization process. Moreover, the incorporation of heavy metals disrupts the C-S-H and N-A(F)-S-H gel structure, leading to increased matrix cracking and reduced compressive strength. However, in Figure 9h, it can be observed that adding PVA-KH792 further improves the matrix cracks, thereby increasing compressive strength.

**Figure 10:**
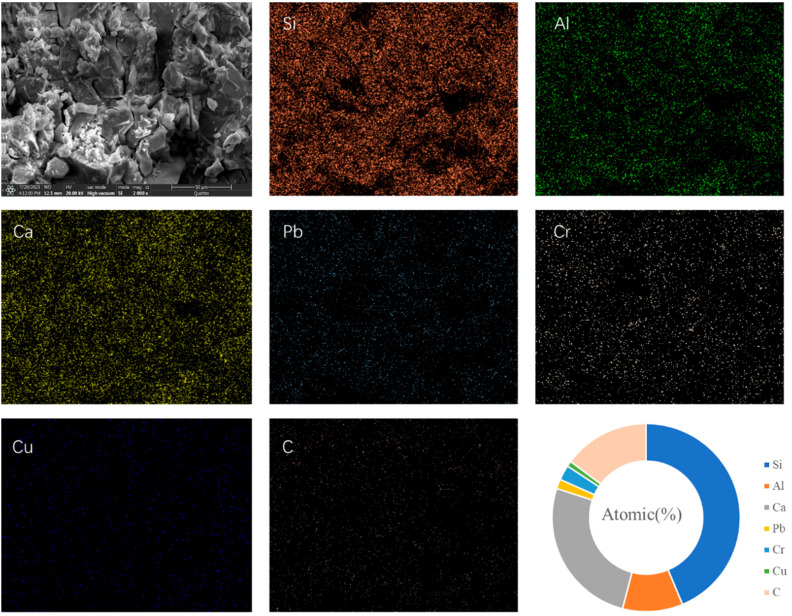
SEM-EDS images of LZEC.

Figure 10 presents the SEM-EDS image of LZES. The EDS analysis shows that Si, Al, and Ca are the main elements in the sample, with relatively low Al content, suggesting that the matrix may primarily consist of C-S-H gel. It can be observed that the sample contains a certain amount of carbon, which may be attributed to the formation of carbonates from alkali metals in the cementitious material in the air. The image reveals that the three heavy-metal elements, Pb, Cr, and Cu, are uniformly dispersed in the cementitious material, indicating that ES can dissolve and disperse well in the LZSS matrix.

**Figure 11:**
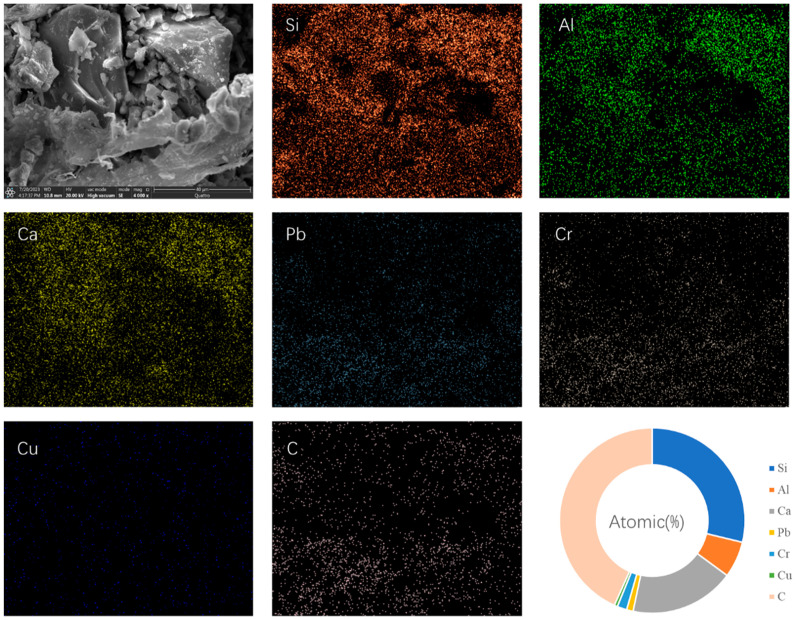
SEM-EDS images of PVA-KH-LZEC.

Figure 11 presents the SEM-EDS image of PVA-KH-LZEC. At the bottom of the image, a thin PVA film is visible, which accounts for the higher carbon content observed in the EDS analysis. In addition, Si, Al, and Ca remain the main components. The ratio of heavy-metal elements remains relatively low. However, compared to the SEM-EDS image of LZEC, the distribution of heavy-metal elements is no longer uniform. Instead, they are more densely distributed in the areas covered by the PVA film. This phenomenon indicates that PVA-KH792 can immobilize some of the heavy metals.

#### 4.1.4. XPS Analysis

**Figure 12:**
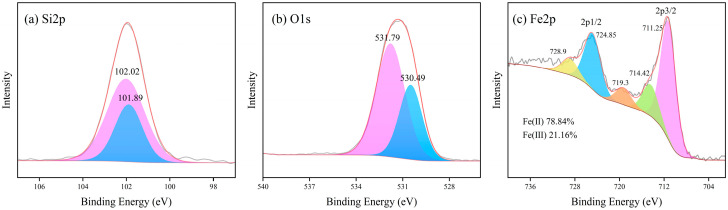
XPS spectra of LZSS: (**a**) Si2p; (**b**) O1s; (**c**) Fe2p.

**Figure 13:**
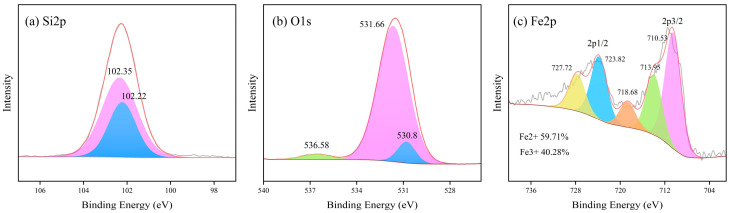
XPS spectra of LZAC: (**a**) Si2p; (**b**) O1s; (**c**) Fe2p.

**Figure 14:**
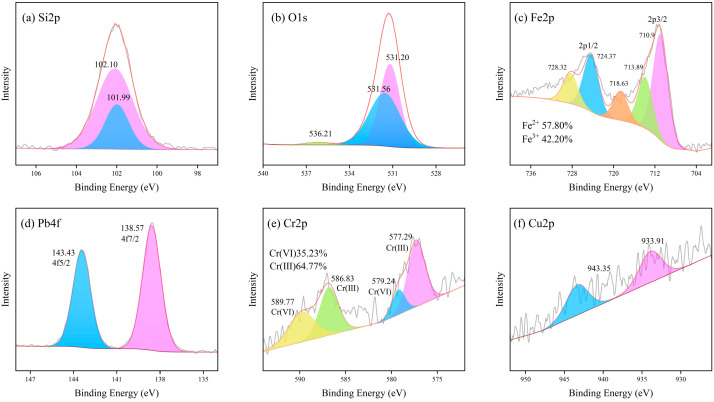
XPS spectra of LZES: (**a**) Si2p; (**b**) O1s; (**c**) Fe2p; (**d**) Pb4f; (**e**) Cr2p; (**f**) Cu2p.

**Figure 15:**
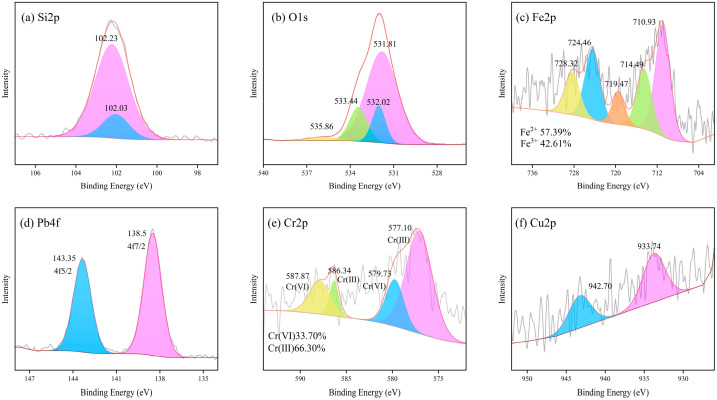
XPS spectra of PVA-KH-LZES: (**a**) Si2p; (**b**) O1s; (**c**) Fe2p; (**d**) Pb4f; (**e**) Cr2p; (**f**) Cu2p.

XPS spectra were obtained for LZSS, LZAC, ES, LZES, and PVA-KH-LZES. As shown in Figure 12a, the Si2p binding energy of LZSS is located at 101.89 and 102.02 eV, corresponding to Si-O-Si and Si-O, respectively [58]. Compared to LZSS, the Si2p binding energy of LZAC (Figure 13a) slightly increases to 102.22 and 102.35 eV, indicating the formation of stable geopolymer gels through the alkaline activation reaction of most active SiO_2_, resulting in a shift in the Si2p peak to higher binding energy [59]. From Figure 14a, it can be observed that the inclusion of ES disrupts the formation of the geopolymer network, leading to a decrease in Si2p binding energy [60]. However, after the addition of PVA-KH792, the Si2p binding energy increases, indicating that PVA-KH792 mitigates the structural damage caused by heavy metals in ES. The O1s spectrum of LZSS (Figure 12b) exhibits two peaks, with one at 530.49 eV corresponding to Fe-O. Due to the overlap of the Si2p 1/2 and Si2p 3/2 peaks, it becomes difficult to distinguish between Si-O and Si-O-Si bonds in the Si-O-Si network [61]. Thus, the peak observed at 531.79 eV in the O1s spectrum of LZSS may correspond to both Si-O-Fe and Si-O-Si bonds. Alkali activation of slag leads to depolymerization and polymerization reactions [62], as evidenced by the weakening of the Fe-O peak and the appearance of a new peak in the O1s spectrum of LZAC (Figure 13b) at 536.58 eV, corresponding to the formation of aluminosilicate byproducts (CaAl2O4 and SiAl2O4) [63]. In the O1s spectrum of LZES (Figure 14b), the Fe-O peak disappears completely, and the peak around 531 eV indicates separation, with the peak at 531.20 eV corresponding to Si-O-Si and the peak at 531.56 eV possibly corresponding to Si-O-M (M = Fe, Pb, Cr, Cu). In the spectrum of PVA-KH792 addition (Figure 15b), a new peak appears at 533.44 eV, possibly due to the presence of PVA. Given the significant Fe content in LZSS, changes in Fe during the alkaline activation process cannot be overlooked. Figure 12c shows that the Fe2p spectrum of LZSS exhibits two prominent peaks near 711 and 724 eV, corresponding to Fe2p 3/2 and Fe2p 1/2, respectively. By deconvoluting Fe2p 3/2 and Fe2p 1/2 using the Gaussian-Lorentz method [64], the peaks at 714.42 and 728.9 eV can be attributed to Fe(III), while those at 711.25 and 724.85 eV correspond to Fe(II) [65]. The peak at 719.3 eV corresponds to the Fe(III) satellite peak [66]. After geopolymerization, the Fe(III) content in LZAC (Figure 13c) increases from 21.16% to 40.28%, indicating that the original Fe(II) species in LZSS dissolve into the alkaline solution and undergo polymerization and recombination, accompanied by the oxidation of Fe(II). Upon the addition of ES, the Fe(II) content in LZES (Figure 14c) decreases from 59.71% in LZAC to 57.80%. These can be attributed to two reasons: (1) the inclusion of ES increases the setting time of the sample, leading to more Fe(II) being oxidized in the air, and (2) during the geopolymerization process, Fe(II) reduces Cr(VI) in ES to Cr(III). Furthermore, after the addition of PVA-KH792, the Fe(II) content decreases slightly again, mainly due to the prolonged hydration time, which exposes the raw materials to air for a longer period, leading to oxidation.

**Figure 16:**
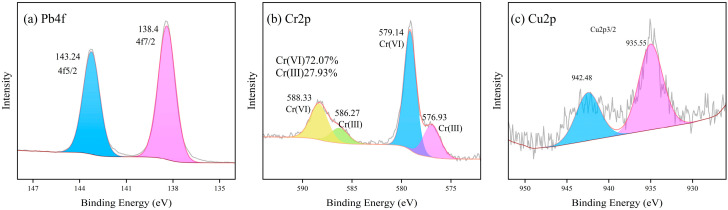
XPS spectra of ES: (**a**) Pb4f; (**b**) Cr2p; (**c**) Cu2p.

ES contains significant heavy metals, predominantly Pb, Cr, and Cu. The XPS spectra of ES are shown in Figure 16. The Pb4f spectrum (Figure 16a) exhibits a pair of peaks at 138.4 and 143.24 eV, corresponding to 4f7/2 and 4f5/2, indicating a monovalent state of Pb in ES. XRD analysis confirms that the main component of ES is lead chromate, suggesting the presence of Pb(II). Similar peaks are observed in the spectra of LZES (Figure 14d) and PVA-KH-LZES (Figure 15d), indicating that the valence state of Pb remains unchanged during geopolymerization. Lead chromate dissolves in an alkaline solution, and compared to ES, the binding energy of Pb in LZES increases from 138.4 and 143.24 eV to 138.57 and 143.43 eV, suggesting that the dissolved Pb is immobilized in the cementitious material. Compared to LZES, the binding energy of Pb in PVA-KH-LZES slightly decreases, possibly due to the partial fixation of Pb through complexation with PVA-KH792. The Cr 2p spectrum of ES (Figure 14b) corresponds to 2p1/2 and 2p3/2, with peaks at 577.29 and 586.83 eV attributed to Cr(III), and peaks at 579.24 and 589.77 eV attributed to Cr(VI) [67]. The proportion of Cr(VI) is 72.07%, while Cr(III) accounts for 27.93%. However, in the spectra of LZES (Figure 14e) and PVA-KH-LZES (Figure 15e), a significant decrease in the proportion of Cr(VI) is observed, with values of 35.23% and 33.70%, respectively, indicating the reduction in Cr(VI) to Cr(III) during the alkaline activation process. This observation suggests that Fe(II) during geopolymerization reduces Cr(VI) to Cr(III), as changes in Cr(VI) content correlate inversely with Fe(III) content. The peaks at 942.48 and 935.55 eV in Figure 16c correspond to the Cu2p 3/2 and its satellite, indicating the presence of Cu(II) in ES [68]. In LZES (Figure 14f) and PVA-KH-LZES (Figure 15f), the binding energies are 933.91 and 933.74 eV, respectively, suggesting that some Cu exists in the form of Cu(OH)_2_. Compared to ES, LZES, and PVA-KH-LZES exhibit higher binding energies for the Cu2p 3/2 peak, indicating better immobilization of Cu and suggesting that Cu may be incorporated into the three-dimensional network structure of the cementitious material.

### 4.2. Alkaline Activation Geopolymerization Process and the Potential Mechanism of Heavy-Metal Immobilization

**Figure 17:**
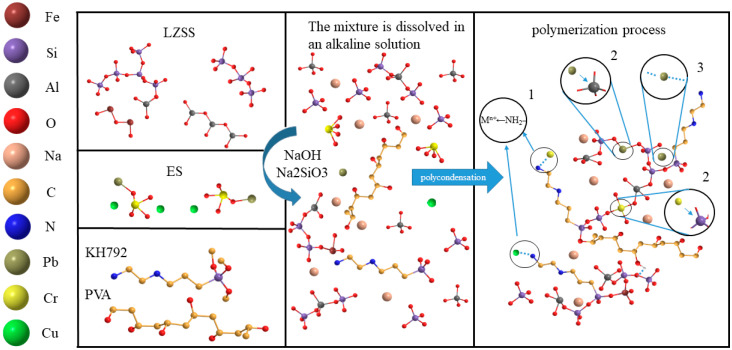
Potential mechanism of alkali-activated geopolymerization process and heavy-metal immobilization.

Based on the abovementioned analysis, the reaction process is illustrated in Figure 17. LZSS, ES, and PVA-KH792 were uniformly mixed and added to the alkaline activating solution. During this process, ES dissolved and released its heavy-metal content, with some Cr(VI) being reduced to Cr(III) by Fe(II). Simultaneously, organic and cementitious materials underwent crosslinking in the presence of KH792. Heavy metals were immobilized in the cementitious material during condensation through various mechanisms.

Based on the combined evidence from XRD, FTIR, SEM-EDS, and XPS, the evolution of compressive strength and heavy-metal immobilization in this system can be interpreted in an integrated manner. First, the XRD results indicate that alkali activation of LZSS generated reaction products dominated by an amorphous gel phase, accompanied by the dissolution and reconstruction of the original mineral phases. This gel framework constituted the basic structural skeleton responsible for both strength development and heavy-metal incorporation. The FTIR results further show variations in the Si-O-T (T = Si or Al) related bands, suggesting the rearrangement of the aluminosilicate network and the formation of a more stable three-dimensional binding structure during geopolymerization. The SEM-EDS observations provide direct microstructural support for this interpretation: compared with the unmodified samples or samples with relatively high ES content, the PVA-KH792-modified specimens exhibited a denser matrix, fewer cracks, and tighter interfacial bonding, indicating that the co-modification alleviated the structural damage caused by ES incorporation and improved the physical encapsulation capacity of the matrix toward heavy metals. In our study, the shift in the Si2p binding energy in XPS (from 102.22 eV to 102.35 eV) and the matrix densification observed in SEM provide physical and chemical evidence of this crosslinking.

More importantly, the XPS results provide chemical-level evidence that supports the proposed immobilization mechanism. On the one hand, the evolution of Fe valence states suggests that Fe(II) participated in the reduction of Cr(VI) to Cr(III), thereby decreasing the mobility and toxicity of chromium. On the other hand, the changes in the binding environments of Pb, Zn, and other heavy metals indicate that these elements were not merely present as free species, but were more likely stabilized within the matrix through gel encapsulation, surface complexation, electrostatic adsorption, and interactions with functional groups. PVA introduced hydroxyl-rich polymer chains, which improved matrix toughness and provided additional interaction sites for metal ions, whereas KH792, after hydrolysis, supplied silanol and amino groups that strengthened the organic-inorganic interface and promoted the fixation of heavy metals within the gel structure. Therefore, the immobilization of heavy metals in this study should be understood as a multi-mechanism process dominated by gel-network encapsulation, dense-structure barrier effects, functional-group complexation/adsorption, and Fe(II)-assisted reduction in Cr(VI). This synergistic mechanism not only explains the improvement in compressive strength observed after PVA-KH792 modification but also corresponds well with the reduced leaching concentrations, demonstrating that mechanical enhancement and heavy-metal stabilization in this system share a common structural and chemical basis.

To quantitatively validate the proposed immobilization pathways, the XPS data serve as a critical indicator. Specifically, the reduction in Cr(VI) to Cr(III) is supported quantitatively by the decrease in the Cr(VI) peak proportion from 72.07% in the raw ES to 33.70% in PVA-KH-LZES. Because this system is highly alkaline due to the activator, the reduction in Cr(VI) by Fe(II) from the LZSS can be represented by the following proposed redox reaction in a basic medium:

(1) $$3Fe{\left(OH\right)}_{2}+CrO_{4}^{2-}+4H_{2}O\to 3Fe{\left(OH\right)}_{3}+Cr{\left(OH\right)}_{3}+2OH^{-}\;\;\;\;\;\;\;\;\;\;\;\;\;\;\;\;\;\;\;\;\;\;\;\;\;\;\;\;\;\;\;$$

Thermodynamically, in an alkaline environment, the reduction potential of the CrO_4_^2−^/Cr(OH)_3_ couple is sufficiently positive relative to the Fe(OH)_3_/Fe(OH)_2_ oxidation, making the spontaneous reduction in highly mobile Cr(VI) to stable, insoluble Cr(III) thermodynamically favorable (∆G < 0).

Furthermore, the interaction between KH792 and the geopolymer matrix can be described by the hydrolysis of the methoxy groups on the silane, followed by a condensation reaction with the surface hydroxyls (silanols) of the aluminosilicate gel:

Hydrolysis:

(2) $$R-Si(OCH_{3}{)}_{3}+3H_{2}O\to R-Si(OH{)}_{3}+3CH_{3}OH\;\;\;\;\;\;\;\;\;\;\;\;\;\;\;\;\;\;\;\;$$

Condensation with matrix:

(3) $$R-Si(OH{)}_{3}+HO-Si(Matrix)\to R-Si-O-Si(Matrix)+H_{2}O\;\;\;\;\;\;\;\;\;\;\;\;\;\;\;$$

where R represents the aminoalkyl functional group of KH792.

The resulting covalent Si-O-Si linkages provide the structural densification observed in the SEM analysis. At the same time, the unreacted functional groups on the PVA and KH792 polymer chains serve as active sites for the complexation of heavy metals such as Pb and Cu, thereby trapping them within the crosslinked network.

## 5. Conclusions

This study aimed to prepare cementitious materials from LZSS via alkaline activation. The influence of the polymers PVA and KH792 on the cementitious material was investigated, and the effects and mechanisms of composite cementitious materials on heavy-metal immobilization were studied using ES as a heavy-metal source. The conclusions drawn from the study, based on the effects of polymer on the mechanical properties of the samples through compressive strength tests, the effectiveness of different samples in immobilizing heavy metals through toxicity leaching tests, and the investigation of the heavy-metal immobilization mechanism through XRD, FTIR, SEM-EDS, and XPS analysis, are as follows:

(1) When PVA or KH792 is added individually, there is a limited increase in the compressive strength of the samples. However, the 0.5% PVA and 0.5% KH792 composite addition outperforms individual additions, indicating crosslinking between PVA and KH792.

(2) The leaching concentration of heavy metals in LZES exceeds the limit when ES content is 5%. However, the composite addition of PVA-KH792 can meet the standard requirements for heavy-metal concentration at 5% ES content.

(3) A portion of highly toxic Cr(VI) is reduced to low toxic Cr(III) by Fe(II) in LZSS and subsequently immobilized.

(4) PVA-KH792 strengthens the immobilization of heavy metals in cementitious materials through adsorption, complexation, and other mechanisms.

(5) Limitations and future perspectives:

While the results demonstrate the feasibility of using LZSS and ES in a PVA-KH792-modified composite cementitious system, several experimental limitations and uncertainties must be acknowledged. First, based on the present mechanical and leaching results, the developed material is more suitably considered for non-structural construction products, such as paving blocks, masonry blocks, and other secondary building units, rather than load-bearing structural applications. Further durability and scale-up studies are required before practical implementation. Second, the proposed polymer-matrix interactions, though supported by XPS, FTIR, and macroscopic performance data, are primarily inferred from ex situ solid-state characterization; advanced in situ techniques, such as solid-state Nuclear Magnetic Resonance (NMR), would be required to definitively map molecular crosslinking. Third, this study evaluates early-age mechanical performance and static leaching behavior under controlled laboratory conditions.

For future work, transitioning this novel material from the laboratory to large-scale engineering applications will require comprehensive durability studies. Future investigations should focus on the long-term immobilization stability of heavy metals under dynamic environmental stressors, including extended wet–dry and freeze–thaw cycling, carbonation, and aggressive sulfate exposure. Additionally, pilot-scale studies considering the variability of raw industrial waste batches and the economic feasibility of utilizing PVA and KH792 at an industrial scale are necessary to validate the sustainability and commercial viability of this approach fully.

## Figures and Tables

**Table 1:** Chemical composition of LZSS.

Fe_2_O_3_	SiO_2_	CaO	Al_2_O_3_	MgO	MnO	TiO_2_	K_2_O	ZnO	Others
38.53	27.31	16.22	7.88	5.21	1.16	0.649	0.6	0.378	2.06

**Table 2:** Chemical composition of ES.

PbO	Cr_2_O_3_	BaO	Fe_2_O_3_	SnO_2_	SO_3_	CuO	Na_2_O	Cl	Others
52.7	16.9	7.51	4.93	4.91	4.35	2.43	2.22	1.68	2.37

**Table 3:** Leaching concentrations and leaching limits of heavy metals in LZSS and ES.

The Leaching Method and Standard	Heavy-Metal Elements				
	Zn (mg/L)	Cu (mg/L)	Pb (mg/L)	Cr (mg/L)	Cr(VI) (mg/L)
LZSS (TCLP)	57.2	4.61	0.34	/	/
LZSS (HJ/T299-2007)	0.82	17.51	0.15	/	/
ES (TCLP)	/	181.28	4.24	353.4	172.68
ES (HJ/T299-2007)	/	53.62	2.92	440.1	248.84
EPA limits	/	15	5	15	2.5
GB5085.3-2007 limits	100	100	5	15	5

**Table 4:** Physicochemical properties of PVA.

Degree of Polymerization	Molecular Weight	Alcoholysis Degree (mol%)	Viscosity (mPa·s)	Volatile (%)	Ash (%)	pH	Purity (%)
1700	22,000	87.0–89.0	20.5–24.5	≤5	≤0.5	5–7	93.5

**Table 5:** Single-factor experimental design table.

Samples	Alkali Content	The Module of Water Glass	Liquid–Solid Ratio
A1	2.5	1.4	0.22
A2	3.0		
A3	3.5		
A4	4.0		
A5	4.5		
S1	3.5	1.2	0.22
S2		1.3	
S3		1.4	
S4		1.5	
S5		1.6	
L1		1.4	0.20
L2			0.22
L3			0.24
L4			0.26
L5			0.28

**Table 6:** Leaching concentration of Zn with different dosages of PVA, KH792, and PVA-KH792.

Samples	TCLP (mg/L)	Sulfuric–Nitric Acid Method (mg/L)
0%PVA	46.31	0.43
0.5%PVA	42.43	0.19
1%PVA	43.92	0.34
1.5%PVA	44.63	0.38
2%PVA	47.58	0.56
0%KH792	46.31	0.43
0.5 KH792	46.21	0.42
1%KH792	45.37	0.35
1.5%KH792	41.76	0.29
2%KH792	41.15	0.21
0.5% PVA + 0.5% KH792	31.03	0.27

## Data Availability

The original contributions presented in this study are included in the article. Further inquiries can be directed to the corresponding author.

## Abbreviations

The following abbreviations are used in this manuscript:

LZSS	Lead–zinc smelting slag
ES	Electroplating sludge
PVA	Polyvinyl alcohol
KH792	[3-(2-Aminoethyl)aminopropyl] trimethoxysilane
